# Manageable N-doped Graphene for High Performance Oxygen Reduction Reaction

**DOI:** 10.1038/srep02771

**Published:** 2013-09-26

**Authors:** Yuewei Zhang, Jun Ge, Lu Wang, Donghong Wang, Feng Ding, Xiaoming Tao, Wei Chen

**Affiliations:** 1 i-Lab, Suzhou Institute of Nano-Tech and Nano-Bionics, Chinese Academy of Sciences, Suzhou 215123, P. R. China; 2 Department of Applied Physics, The Hong Kong Polytechnic University, Kowloon, Hong Kong SAR, P. R. China; 3 Institute of Textile and Clothing, The Hong Kong Polytechnic University, Kowloon, Hong Kong SAR, P. R. China

## Abstract

Catalysts for oxygen reduction reaction (ORR) are at the heart of key green-energy fuel cell technology. N-doped graphene is a potential metal-free electrode with much better electrocatalytic activity, long-term stability, and tolerance to crossover effect than expensive platinum-based electrocatalysts. Here, we report a feasible direct-synthesis method in preparing N-graphene with manageable N contents in a large scale. The resultant N-graphene used as electrocatalysts exhibits similar catalytic activity but superior stability compared to commercial Pt/C for ORR in an alkaline solution. It was found that their electrocatalytic activities were demonstrated to depend largely on N-doping content. When nitrogen content reaches a high value at about 24–25%, ORR reaction exhibits a favorable formation of water via a four-electron pathway. Furthermore, the effect of pyrolysis temperature and precursor on the activity of N-graphene is systematically analyzed, and may shed some light on the principle of choosing appropriate way for preparing N-graphene.

Fuel cells, which offer a highly efficient and fuel-flexible technology that cleanly produces power and heat with a multitude of end-uses, hold potential to dramatically impact clean energy economy. The main obstacle to the development of fuel cells lies in the sluggish oxygen reduction reaction (ORR)^1^. Nowadays, Pt and its alloys have been recognized as the most effective catalysts for ORR^2^. However, the platinum-based electrocatalysts are scarcely available at a high price and suffering declining activity. Owing to this fact, ongoing research is focused on identifying and developing alternative materials that will reduce the cost and extend the life of fuel cell.

Recent years, graphene draws much attention since it is a fantastic material with many superlatives^3^,^4^,^5^. Since the first report in 2004^6^, tremendous work has been carried out to apply graphene into various applications by adjusting its physico-chemical property. Chemical control is an effective approach to tailor the property of graphene^7^,^8^,^9^,^10^,^11^. The most widely introduced heteroatom is nitrogen, which has been proved in many nitrogen-doped carbons, such as N-doped carbon nanotube^12^, colloidal graphene quantum dots^13^, carbon sub-micrometer spheres^14^. N-graphene, which behaves different from the pristine graphene, has been explored for applications in Li-ion batteries, biosensors, ultracapacitors and photocatalyst, especially fuel cells^15^. N-graphene has been reported to possess long-term durability and tolerance to poisoning as metal-free catalysts^16^. Most importantly, it has considerable electrochemical activity for ORR^16^,^17^,^18^.

N-rich graphene has been realized mainly through two different ways: one of them is direct synthesis, such as chemical vapor deposition (CVD)^19^, segregation growth approach^20^, solvothermal^21^. Another is post treatment, such as thermal treatment in NH_3_^7^, plasma treatment^22^ and hydrazine hydrate^23^. However, most of these methods cannot succeed in homogeneous nitrogen doping with high nitrogen content. The doping level reported is generally less than 20%.

Nitrogen atoms in CN heterocyclic ring can influence the spin density and charge distribution of neighbor C atoms and activate them. Judged from reported articles, the doping content and type are closely related to ORR activity for N-graphene. Generally, N-graphene consists of three nitrogen types, as pyridinic N, pyrrole N and graphitic N. How the different N decides the ORR activity is still controversial due to lack of experimental results. Apart from the N types, electrochemical activity is strongly influenced by nitrogen content. Lower N content is unable to render enough N-doped graphene electrochemical activity. Higher N content is capable of creating more active sites, however it can also result in low conductivity and more sites prone to poisoning. Thus, graphene with suitable N content which maintains acceptable conductivity and considerable active sites is desirable. Developing a method to manage nitrogen doping in a large scale is critical to figure out the optimal nitrogen content for ORR. Therefore, we try and succeed in preparing manageable N-graphene with N content up to 33%, and discover optimized ratio of C/N (~3) for ORR through plenty of experimental results.

## Results

Here, we report a two-step facile way to synthesis N-doped graphene starting from a reactive graphitic carbon source (*e.g.* glucose) and N rich molecules (*e.g.* melamine and urea). Glucose is an appropriate candidate since it is a single sugar which distributed in nature widely and undergoes carbonization at high temperature. Moreover, glucose is the hydrolysis product of sucrose which has been proved effectively in transforming into carbons^24^. As for N rich molecules, melamine is chosen for its ability to evolve into two-dimensional graphene-like graphitic carbon nitride (g-C_3_N_4_)^25^,^26^. In our previous work, we also explored porous g-C_3_N_4_ simply polymerized from urea in air without extra templates^25^,^27^. Laminar g-C_3_N_4_ can act as templates for the formation of 2D structure as well as nitrogen source^28^. In this work, N-doped graphene with tunable content and types of N atom can be obtained. Thus further investigation of the dependence of ORR activity on the N doping can be realized. Nitrogen-doped graphene samples were prepared from a two-step approach. The processes are illustrated in Figure S1. Glucose and another component were mixed uniformly first, then heated in muffle furnace for pre-treatment and then in tube furnace for deeper treatment. The final products were denoted as NCX_yz_ (Table S1). X is the first letter of urea or melamine. y = 5 represents the temperature (T1 = 550°C) in muffle furnace. z ( = 7, 8, 9) represents the temperature (T2 = 700, 800, 900°C) in tube furnace.

### Characterization of N-graphene

**Figure 1:**
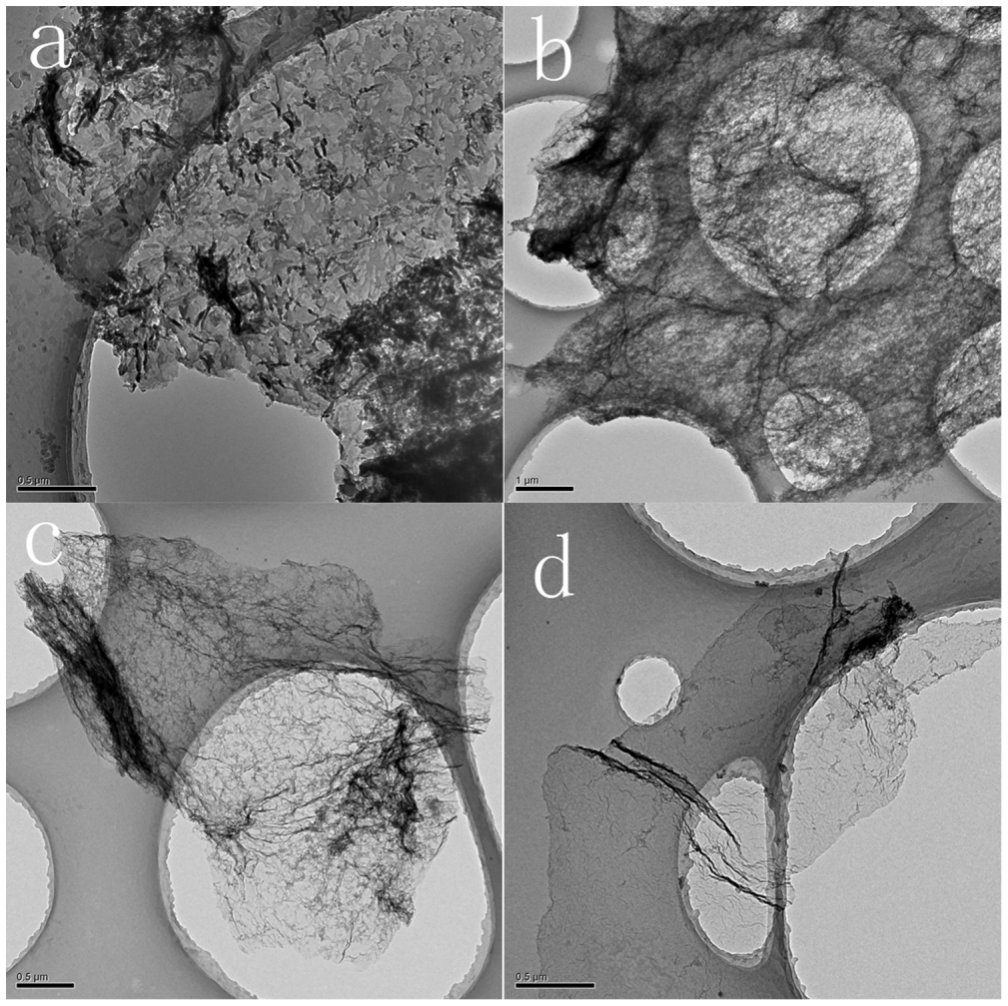
Typical TEM images of N-doped graphene. (a) NCM_5_, (b) NCM_57_, (c) NCM_58_, (d) NCM_59_. Scale bar is 0.5 μm for (a, c, d) and 1 μm for (b).

The as-prepared products were investigated by transmission electron microscopy (TEM). It can be seen that after the first step, a large and thick two dimensional structure forms (Fig. 1a). After treated with second step, products tend to consist of fewer layers. Samples with T2 = 700°C have obvious pores and wrinkles on the edge. Along with higher T2 temperature, we can succeed in smoother sheets with fewer wrinkles (Fig. 1b–d), indicating recovery of graphitic carbon structures. In addition, no obvious morphology difference is detected when urea replaces melamine (Fig. S2a–d).

The scanning electron microscopy (SEM) can also provide more morphology details about lamellar aggregates. Fig. S3c–d show a loose lamellar and silk-like structures for NCM_58_ and NCU_58_, while the one-step products reveal a compact coral-like structure as g-C_3_N_4_ (Fig. S3a–b).

**Figure 2:**
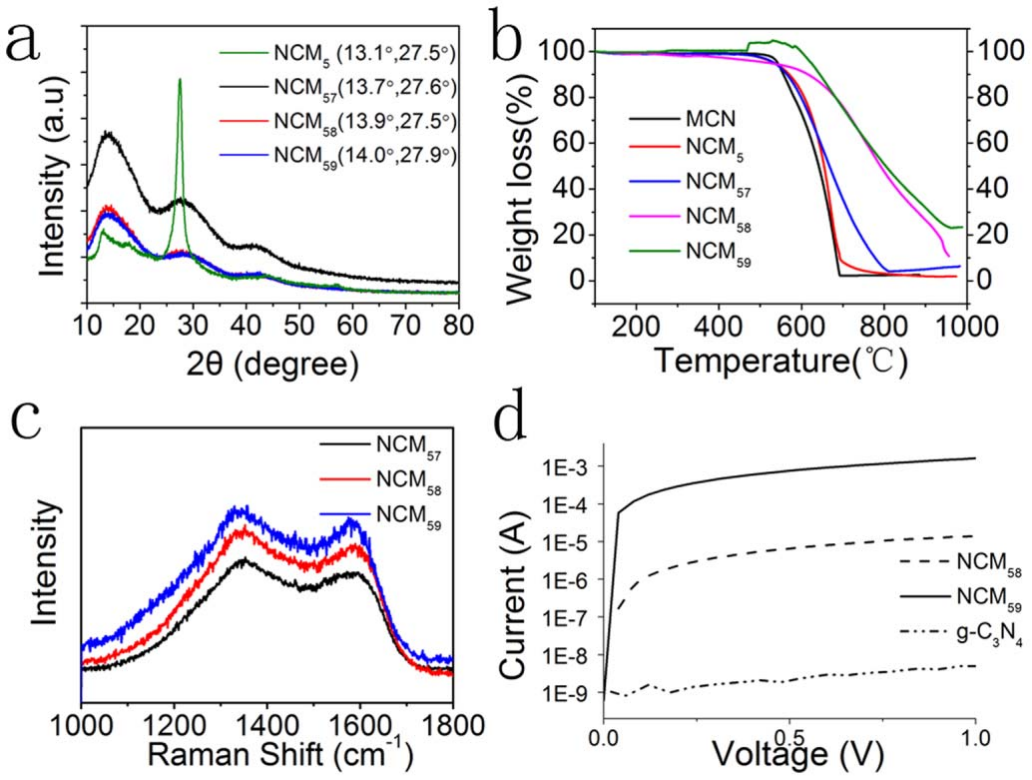
Structure characterizations of N-graphene derived from glucose and melamine. (a) XRD patterns. (b) Thermo Gravimetric Analyzer (TGA) measurements. (c) Raman spectra of different samples. (d) I-V plots.

Layered structure can also be proved by X-ray diffraction (XRD) patterns. As shown in Fig. 2a, XRD pattern of NCM_5_ with two peaks at 2θ = 13.1° and 27.5° is similar to g-C_3_N_4_, suggesting that the material is made up with g-C_3_N_4_ mostly. The peak around 27.5° corresponds to interlayerspacing (d = 3.24 Å). After NCM_5_ undergoes further treatment, XRD patterns have a dramatically obvious change. The intensity of the peak around 27.5° gradually becomes much weaker, while the peak around 14° gets much stronger. Since melamine derived carbon nitride cannot exist above 700°C according to theThermo Gravimetric Analyzer (TGA) measurement result (Fig. 2b), it is assumed that the peak around 14.0° of NCM_57,8,9_ belongs to N-doped graphene, corresponding to lamellar distance d = 0.63 nm. The distance is almost twice larger than pure graphene. According to the former discussion, NCM_5_ contains mostly graphitic carbon nitride, which will decompose above 700°C. A large amount of gas will release during its decomposition. After melamine derived structures disappear,interlamellarspacing is enlarged, and glucose-obtained products preserve lamellar structure. Enlarged space is in favor of mass transfer. When applying urea as a precursor, the polymerization of urea is largely hindered, thus the lamellar structure ordering of NCU_5_ is decreased. Followed by second-step treating, the final result as NCU_59_ exhibit a corresponding disorder structure which has two apparent peaks rather than one peak in NCM_59_ (Fig. S4a).

Thermo Gravimetric Analyzer (TGA) results show that pure melamine derived carbon nitride (MCN) decomposes completely before 700°C. As for NCM_5_, huge mass loss happens before 700°C as well, which means that NCM_5_ constitutes unstable carbon nitride mostly. After treating with high T2, thermal stability of the products is much enhanced, indicating stable C-C bonds accounts for more proportion with increased temperature. About 23% mass of NCM_59_left even conducting at 1000°C in N_2_ flow. The same phenomenon can be observed in the NCU_5_ series (Fig. S4b).

Fig. 2c shows the Raman spectra for N-doped graphene. The D band around 1350 cm^−1^ is known as the disorder band resulting from defects or edges in the graphene. The peak near 1580 cm^−1^ is the main spectral feature of graphene which derived from in-plane motion of the carbon atoms. We can see that the I_D_/I_G_ ratio becomes slightly lower when the powders are treated with higher T2, which indicates the recovery of structural distortion induced by heteroatom interference. When we replace melamine with urea, the ratio is changing the same way as shown in Fig. S4c.

Graphene is known as excellent conducting medium. However, the addition of N can destroy its carbon structure and reduce its conductivity. The conductivity of N-doped graphene samples is tested using I-V (Fig. 2d). The measured resistance of NCM_7_ is about 10^8^ Ω, as large as that of g-C_3_N_4_, although its N percent has dropped to 30%. NCM_58_ shows improved electrical properties with resistance around 10^5^ Ω. NCM_59_ is a better electron transfer media with resistance around 10^2^ Ω, which is reasonable due to its recovered graphitic carbon structure and higher carbon content we will discuss later.

Judging from above results, this two-step synthesis approach with different precursors can lead to similar two-dimensional graphene like structure. Melamine or urea can polymerize into 2D carbon nitride and provide template for glucose although it decomposed finally. Thin few-layer structure and excellent conductivity can be obtained finally.

**Figure 3:**
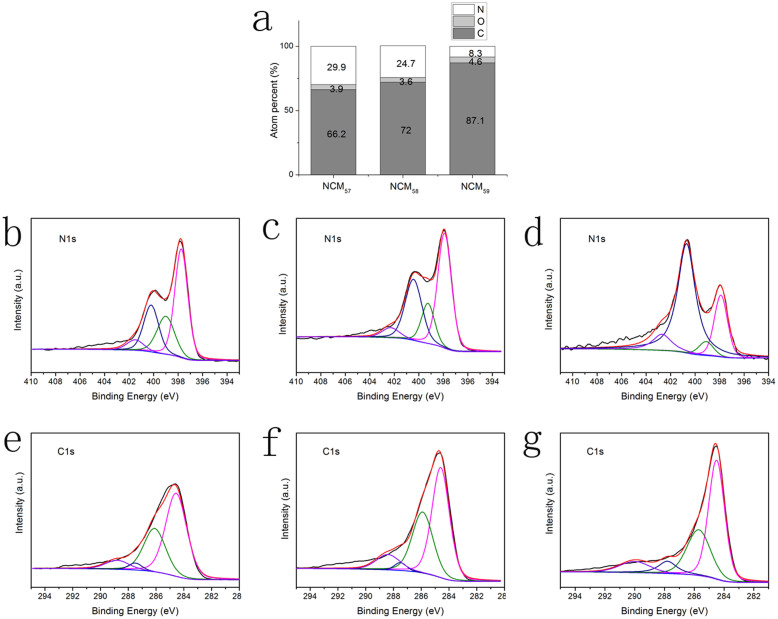
Elemental analysis of NCM_57_, NCM_58_ and NCM_59_. (a) The content of C, N and O in three samples. High resolution N1s XPS spectra of (b) NCM_57_, (c) NCM_58_ and (d)NCM_59_. High resolution C1s XPS spectra of (e) NCM_57_, (f) NCM_58_ and (g) NCM_59_.

Furthermore, the addition of N-enrichment precursor melamine can act not only as template, but also as N source for glucose-derived products. To confirm N in the lamellar sheets, we examine chemical compositions and element binding state using a powerful tool–X-ray photoelectron spectroscopy (XPS).The element composition from XPS spectra can be seen in Fig. 3a. N atoms take over a large part of total atoms. With 41.77% C atoms and 55.75% N atoms, the C/N ratio ofNCM_5_ (0.75) is equal to that of g-C_3_N_4_ (0.75). In Fig. S5, the XPS spectra show that N spectrum of NCM_5_ possesses typical g-C_3_N_4_characteristic, which can been separated into four peaks as reported^25^. Along with previously mentioned similar XRD patterns of NCM_5_ and g-C_3_N_4_ earlier, we believe NCM_5_ is mainly made up with melamine-derived carbon nitride. When materials go through second-step treatment, and T2 temperature goes up, nitrogen content varies from 55.8% to 8.3%. The C/N ratio increases from 0.75 to 10.5 remarkably according to XPS results (Fig. 3a), which is in line with EDX measurement (Fig. S6).

Speaking of urea series as listed in Fig. S8, the change trend follows the same as the NCM_5_ series. However, the C/N ratio of UCN_5_ is 1.4, with only 39.9% of N. In Fig. S7, the spectra of NCU_5_ are quite different from that of g-C_3_N_4_. In consideration of the previous XRD results, it is reasonable to judge that UCN_5_ contains part of incomplete polymerized urea and glucose derived product. As the temperature rises, N atoms also reduce, and the nitrogen content for NCU_57_, NCU_58_, NCU_59_ is 33.1%, 24.1% and 11.2%, respectively (Fig. S8). Nitrogen content measured by EDX also provides similar results (Fig. S6).

Besides tunable N content, we succeeded in preparing samples with variable nitrogen doping states. The N1s spectrum is usually used to determine the nitrogen configurations. The N1s spectrum of NCM_5_ series can be fitted into four peaks at around 397.7, 399.0, 400.2 and 401.5 eV, corresponding to pyridinic N, pyrrolic N, graphitic N and oxygenated N (Fig. 3b–d). We sum up the ratio of separate N state in different types. Interestingly, although NCM_58_ suffers higher mass loss from NCM_5_ than NCM_57_, the ratio of pyridinic N to graphitic N changes little. The pyridinic N occupies 48% in total N atoms of NCM_57_ and NCM_58_. Only after heating NCM_5_ under 900°C, graphitic N is superior in numbers apparently, and exceeds that of pyridinic N. Pyrrole N accounts a small amount in total N. High resolution C1s spectrum can be separated into four peaks too. The peaks centering at around 284.5, 286.0, 287.5 and 288.5 eV corresponding to graphitic carbon (C–C), C-O/C=N, C=N/C-O and O-C=O respectively (Fig. 3e–g). The C1s spectra change accordingly when nitrogen atoms are doped into carbon structures. The ratio of graphitic carbon in all the types of C bonds increased gradually, suggesting the recovery of graphitic carbon structure. In NCU_5_ series, the same tendency can be seen. Elemental analysis and binding state of NCU_5_ series can be seen from Fig. S8 & 9.

Uniform bulk-heteroatom doping is critical in performing high effective catalytic activity. The energy-dispersive X-Ray mapping images of scanning electron microscopic given in Fig. S10 reveal the uniform distribution of C, N and O atoms in the carbon framework. The outline is consistent with the corresponding SEM images. We believe large-scale homogeneously nitrogen doped graphene materials have been produced with direct two-step approach.

Thus it is apparent that uniform N-graphene with adjustable doping state and content of N can be achieved using this facile and direct synthesis approach. The addition of urea or melamine is able to provide a two-dimensional template for glucose-derived products, which prevents glucose transforming into carbon spheres and expand interlamellarspacing as well. Moreover, they can act as N source. Since we found that no matter which N-rich precursor is applied, the final products possess almost the same element content and chemical environment. Thus this can be seen as a general method to prepare N-graphene.

### Electrochemical tests of N-graphene samples

Tremendous efforts have been put on pursuing replacements for noble-metal Pt to catalyze the oxygen reduction reaction in fuel cells. Metal-free N-doped graphene is a potential ORR catalyst candidate. A lot of research papers have tried to explain the mechanism of the high electrocatalyticactivity of N-doped graphene^15^,^18^,^29^,^30^. One of the most controversial subjects lies in the N content and doping state.

Here, the electrocatalytic activity of N-doped graphene for ORR was first examined by conventional three electrode cyclic voltammetry (CV) in 0.1 M KOH solution with N_2_ or O_2_ saturated. For comparison, Pt/C (20% wt. Pt on Vulcan XC-72R) and pure graphene samples were also tested for comparison under the same condition. As shown in Fig. S11, featureless voltammetric currents within the potential range from 0.2 to −1.0 V for samples treated for NCM_57_, NCM_58_,NCM_59_ and graphene are observed in the presence of N_2_. When T2 equals 700°C, CV shows irregular shape, which may link with low capacitance, resulting from high content of nitrogen. The quasi-rectangular shape of NCM_58_ is due to a supercapacitor effect. When T2 increased to 900°C, CV tends to be more close to a rectangle as that of full-carbon materials.

**Figure 4:**
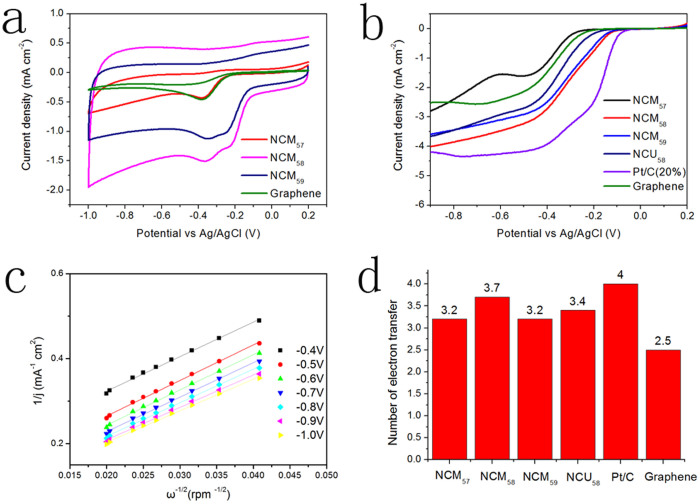
Electrocatalytic activities measurement for NCM_5_ series, NCU_58_, graphene and Pt/C (20%). (a) CV in O_2_ saturated 0.1 M KOH solutions. (b) Rotating Ring-Disk Electrodes LSV at 1600 rpm. (c) Koutecky-Levich plots at different potential for NCM_58_. (d) Electron-transfer numbers at −0.9 V.

In contrast, in the presence of O_2_, a well-defined cathodic peak centered around −0.36 V of NCM_58_ emerges in the CV arising from the oxygen reduction reaction. A similar cathodic process at about −0.36 V was seen for the ORR at other N-doped graphene samples (Fig. 4a). Moreover, all tested sample electrodes displayed dramatic increase in voltammetric currents in O_2_ saturated solutions compared to those of N_2_ saturated solutions, hence suggesting a pronounced electrocatalytic activity of tested N-doped graphene for oxygen reduction. In addition, we can clearly tell that samples obtained at 800°C (the ratios of C/N are around 3) owns much larger voltammetric currents, indicating its better electrochemical activity.

To gain further insight into the oxygen reduction reaction in N-doped graphene, the reaction kinetics were studied by rotating ring-disk electrode (RRDE) voltammery in O_2_ saturated 0.1 M KOH electrolyte at a scanning rate of 10 mV s^−1^. Pt/C (20% wt. Pt on Vulcan XC-72R) and graphene were also tested for comparison under the same condition. Linear sweep voltammetric (LSV) was performed in an O_2_ saturated 0.1 M KOH electrolyte with a rotation from 600 to 2500 rpm. LSV shows that limiting current density increases with higher rotation speeds (Fig. S12). The phenomenon can be explained by the shortened diffusion distance at high speeds, which is in accordance with other studies^31^,^32^. At the 1600 rpm rotation speed, the current density follow the order of NCM_57_< NCM_59_<NCM_58_. At −0.9 V, the current density of NCM_58_ is close to commercial Pt/C (20% wt. Pt). The onset potentials of NCM_58_ electrodes were determined to be at −0.13 V, which is only slightly negative compared to that of Pt/C. ORR performance in the diffusion and kinetically limited regions were evaluated using Koutecky-Levich (K-L) plots as shown in Fig. 4c and Fig. S12 (e–f)as well. All of the K-L plots of RRDE curves from different catalysts show a linear relationship between 1/j and ω^−1/2^ from −0.4 V to −1.0 V. According to Eqa (S1), the number of electrons transferred in the ORR process can be analyzed. The n values for samples were increased as the applied voltage rose. At the potential of −0.9 V, n of NCM_58_ reaches 3.7, indicating that NCM_58_ follows an almost four-electron transfer pathway and has better ORR activity.

**Figure 5:**
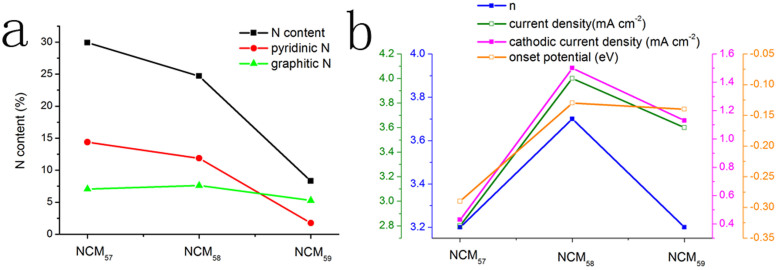
ORR dependence. (a) Content curves of total N, pyridinic N and graphitic N and (b) ORR activities at −0.9 V for NCM_57_, NCM_58_, NCM_59_.

Unlike N-graphene, pure graphene electrode exhibited a two-step, two-electron process for oxygen reduction with the onset potential of about −0.26 V. The catalytic current density was found to be 40% less than that of NCM_58_ over a large potential range. The transferred electron number per oxygen molecule at the graphene electrode was to be 2.5 at the potential of −0.9 V. These results indicate that pure graphene is not ideal for ORR. It is obvious that the doping of N atoms in the carbon ring plays a key role in improving the catalytic activities. Interestingly, from Fig. 5, we can clearly find that NCM_58_ shows highest ORR catalytic activity when taking number of transferred electrons, current density, cathodic current density and onset potential into consideration. In CV tests, although NCM_57_ and NCM_59_ have a cathodic peak at around −0.38 V and −0.34 V respectively, their current density is apparently inferior to that of NCM_58_. The cathodic peak current density is only about one-third for NCM_57_ and two-thirds for NCM_59_ compared to NCM_58_. Moreover, the most positive onset potential of NCM_58_ is another symbol of its higher ORR activity. LSV curve obtained for NCM_58_ of the same mass deposited on RRDE electrode shows current density of 4 mA cm^−2^, much bigger than NCU_57_(2.8 mA cm^−2^) and NCU_59_ (3.6 mA cm^−2^)at −0.9 V. The number of electrons transferred in the ORR reactions based on Koutecky-Levich (K-L) plots (n = 3.2) are also comparatively lower than NCM_58_. When we study the ORR activity of NCU_5_ series, we also find NCU_58_ sample presents better ORR performance (Fig. S13).

To understand the exact dependence of ORR activity on the property of N-graphene, we analyze doping type and content of N atoms more carefully. According to the XPS measurement, N content follows the same change tendency as T2 increases regardless of the CN precursor. The nitrogen gradually decreased as NCM_57_ (30%) > NCM_58_ (25%) > NCM_59_ (8%) and NCU_57_ (33%)> NCU_58_ (24%) > NCU_59_ (12%). Only about 10% nitrogen reserved in all materials treated under T2 = 900°C. Coincidently, although we change the precursor, best electrocatalysts all have the similar N content (~25%) and similar ratio of C/N (~3).

Actually, the variation of N content can result in distinct electrical and electrochemical properties as reported. For instance, graphene with full carbons is a perfect electron transfer medium. However, it cannot act as perfect catalyst due to lack of active sites. Carbon nitride with low conductivity is not appropriate too. In this work, when samples treated under 700°C, carbon structure is not well recovered. Thus although a large amount of N can create active sites, poor electron transfer ability limits its activity. For those treated under 900°C, although carbon structure has been proved to be reconstructed from former morphology and propertiescharacterization, lack of adsorptionsite and active sites hinder its application in ORR. Thus in this work, we prove the composition-dependent ORR activity of the enhanced ORR activity, and a relatively high N content around 25% is a proper value.

Besides the quantity of N atoms, preferred doping type causes another controversy. From reported work, N dopant can result in increased electron density and electron donating properties. Lin, etc. obtained pure pyridinic N doped graphene, and found that pyridinic N efficiently changed the valence band structure of graphene, including the raising of density of π states near the Fermi level and the reduction of work function. However, pyridinic-N is not an effective promoter for ORR activity of carbon, as evidenced by the sluggish activity as reported^33^. The relative electronegativity of graphitic N atoms reduces the electron density on the adjacent C nuclei, and N back donates electrons to adjacent C p_z_ orbitals. The donation and backdonation processes not only facilitate O_2_ dissociation on the adjacent C atoms, but also help form a strong chemical bond between O and C^21^.

Herein, we analyze the relationship between ORR activity and N-doping type. N1s spectra are fitted into four peaks in Fig. 3. Significant change for composition ratios of each type of N species happens when T2 goes from 800°C to 900°C (Fig. S14). Pyridinic N notably decreases which indicates that pyridinic groups are thermally unstable. Graphitic N is finally outnumbered. However the number of graphitic N over pyridinic N did not lead to higher ORR activities. Since dramatically different doping states of nitrogen atoms goes along with decreasing nitrogen content between NCM_58_ and NCM_59_, it is hard to judge which factor plays a more important role here. When comparing NCM_57_ and NCM_58_, little change happens for the ratio of each N type. However, NCM_58_ exceed in ORR properties largely. Thus, we assume that the combination of suitable N content and N doping state will show a synergistic effect in enhancing ORR activities. It is worth noting that when we check the three samples prepared from urea, the dependence of ORR activities on N content and N types follow the same rule.

Actually in the above discussion, we mainly focused on the influence caused by T2, which is one part of the reaction condition. Another important element is the N-rich precursor. Nitrogen enrichment precursor is known as a critical factor in affecting physicochemicalproperty of graphitic carbon nitride. We also conduct experiments on NCU series. ORR electrochemical activities are shown in Fig. S15–16.When taken structure, element composition, the relationship between N doping states and ORR activities into consideration, no obvious difference can be detected. However, the electrochemical activities for each pair of samples treated under the same reaction temperature (Fig. 5 & S13), three samples of NCM_5_ series obviously has larger limiting currents, more positive onset potential and larger number of electrons transferred. Melamine is more preferred for preparing N-doped graphene for ORR. This may attribute to more regular lamellar structure of NCM_5_ series.

**Figure 6:**
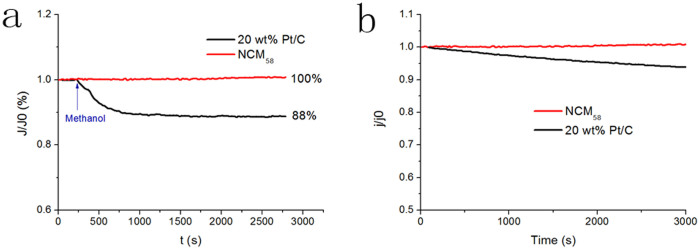
Long time stability and tolerance to alcohol poisoning. (a) The relative ORR cathodic current-time response (1600 rpm) at NCM_58_ and Pt/C (20%) electrodes at −0.5 V in O_2_ saturated 0.1 M KOH before and after adding methanol (3 M). (b) The relative ORR cathodic current-time response (1600 rpm) at NCM_58_ and Pt/C (20%) electrodes at −0.5 V in O_2_ saturated 0.1 M KOH for 3000 s.

To investigate the stability of N-doped graphene electrode toward ORR, we performed chronoamperometric tests of NCM_58_ and Pt/C (20%) electrodes at −0.5 V in O_2_ saturated 0.1 M KOH (Fig. 6a). No obvious loss of current density happens along with the increasing time for NCM_58_. In contrast, the current density of Pt/C (20%) decreases continuously. We also test the poison effect in the presence of fuel molecules (e.g. methanol). The corresponding current-time (i-t) chronoamperometric response for Pt/C electrode given in Fig. 6b shown a sharp decrease in current upon the addition of 3.0 M methanol. However, NCM_58_ maintains its current, suggesting its tolerance to alcohol fuel.

## Discussion

**Figure 7:**
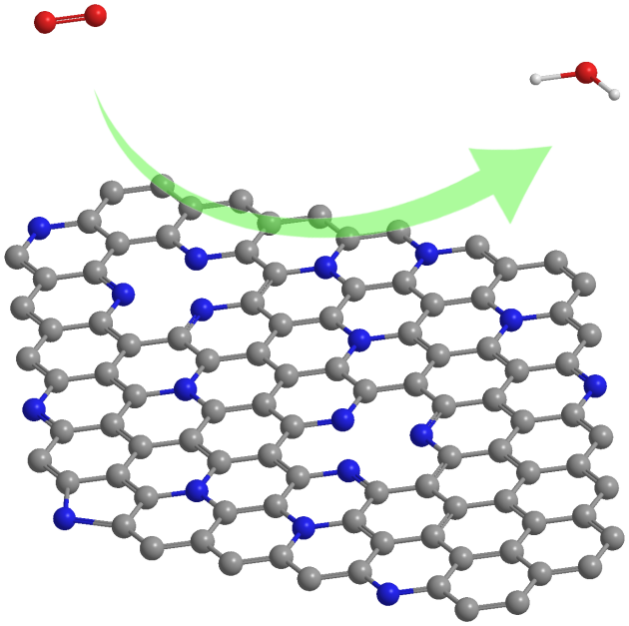
The formal scheme of oxygen reduction on N-graphene. The blue, grey, red, white atoms refer to N, C, O and H respectively.

**Figure 8:**
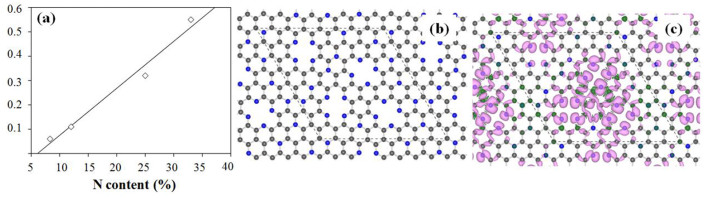
Calculated theoretical results. (a) The percentage (the number of C atoms with a charge density larger than 0.15 divided by the total number of C atoms in the graphene) as the function of N content in graphene; (b) structural model for the N-doped graphene with the N content of 25% (corresponding to the NCU_58_ sample); (c) the spin density distribution on the electron density isovalue plane corresponding to the model in (b), and the most positive value is purple while the most negative value is blue.

There are two types of bonding in graphene: σ-bonding and π-bonding. While the σ-bonding is assumed to be a rigid honeycomb framework built out of two-center two-electron C-C σ-bonds, the π-bonding is supposed to be delocalized. The previous study has proved that aromaticity in graphene is local with two π-electrons located over every hexagon ring^34^. When N doping is introduced, some C atoms are replaced by N and some defects are introduced at the same time, which results in the structural distortions in graphene (see Fig. S17). From quantum mechanics, nitrogen atoms doped in graphene can act as electron acceptor sites, thus nitrogen doping creates an electron acceptor state in the conduction band near the Fermi level^35^,^36^, which destroys the perfect aromaticity of pure graphene. It is reported that the electron-accepting ability of the nitrogen atom will make adjacent carbon net positive, resulting in redistribution of spin density and charge density around the nitrogen atoms, which will influence oxygen adsorption and further electrochemical reactions around the nitrogen atoms^37^ (Fig. 7). Previous studies have shown that the atomic spin density and charge transfer determine the catalytic capability of materials for ORR^38^,^39^. Spin density may be regarded as a factor determining positional selectivity of radical adsorption, while charge density determines the attractive force between charged atoms. We have constructed CN heterocyclic model structures with two types of N (graphitic N and pyridinic N) and different N content (8%, 12%, 25% and 33%). Graphitic N and pyridinic N are expected to be responsible for oxygen reduction activity. We have calculated the spin and charge density in Fig. 8 and Fig. S17. For the charge transfer, it has been proved that the carbon atoms with charge transfer larger than 0.15 are most likely to serve as catalytic active sites^39^, then we have summarized the percentage of C atoms with large charge density (more than 0.15) as function of N content in graphene and shown in Figure 8a. It is found that the active sites of C atoms increase linearly as the N content in graphene increases and the maximum value of charge transfer also increase with the N content from 0.16 for 8% to 0.44 for 33% (see Figure S17). Thus, the samples of NCU_58_ and NCM_58_ with the N content of 25% have more catalytic reactive sites than NCU_59_ and NCM_59_, and exhibit relatively better catalytic capability for ORR, which is consistent with our experimental observations. The spin density analysis has also proved that the NCU_58_ sample with N content of 25% is better with high positive spin density distribution and localized (see Figure 8b–c). It is worthy to note that, for the NCU_57_ sample with high N content of 33%, it has large charge transfer but low catalytic capability. This can be explained in the aspect of its conductivity. For the N-doped graphene with high N content, its conductivity will significantly reduce, thus greatly affect the catalytic capability, which is also observed in a previous study of BCN graphene.

N-graphene is a promising metal-free ORR catalyst with much elusiveness of its electrochemical activity dependence on N doping. In this work, we successfully develop a method to synthesize N-doped graphene with tunable nitrogen atoms in a large content scale. After conducting a lot of contrast measurement, we come to the conclusion that when N content reaches an optimum value as 24 ~ 25% (the ratio of C/N ~ 3), N-graphene shows comparable ORR activities with commercial Pt/C and much better fuel crossover resistance and long-term stability in alkaline medium. Furthermore, melamine is a better N-rich precursor compared to urea for preparing N-graphene as ORR catalyst. Actually, we also find that by pre-treating mixture under 500°C can make as-prepared products prefer a four-electron pathway. Thus it is believed that this approach may offer numerous possibilities in tailoring the physicochemical properties. Since we use a cost-effective and fruitful strategy, it is expected to broaden manageable N-graphene beyond fuel cell application.

## Methods

### Synthesis of N-graphene

N-doped graphene was synthesized by thermal treatment of 0.5 g glucose (from Sigma Aldrich) and 10 g urea(AR, Sinopharm Chemical Reagent Co., Ltd). Firstly, all the precursors were dried at 80°C for 24 h. Then, the precursors were grinded uniformly with a mortar and put in a crucible with a cover. After that, the crucible was placed in Muffle Furnace (Isotemp Programmable Muffle Furnace 650–750 Series, Fisher Scientific) and heated to 550°C for 3 hours under ambient pressure in air respectively. In a typical process, the as-prepared samples were transferred to tube furnace for further high temperature treatment under a protective flow of 200 sccm Ar. In this work, melamine was also used to as a substitute for urea. The final products were denoted as NCX_yz_. X is the first letter of urea and melamine. y represents the temperature (T1 = 550°C) in Muffle Furnace. z ( = 7, 8, 9) represents the temperature (T2 = 700, 800, 900°C) in a furnace tube.

### Synthesis of g-C_3_N_4_

Pure g-C_3_N_4_ is prepared following the process reported. They are denoted as UCN and MCN when applying urea and melamine as precursors respectively.

### Synthesis of graphene

Graphene oxide (GO) was synthesized from natural graphite flake (325 mesh, 99.8%, ABCR GmbH & Co. KG) by a modified Hummers method and has been described in our recent work^40^. The obtained GO solution was sonicated for 30 minutes with 200 W horn digital sonic dismembrator in a program of 2 s on and 5 s off in ice water bath and then reduced with N_2_H_4_^41^. After centrifugation, filtration and washing with deionized water, reduced graphene oxide wet sample were obtained.

### Characterization

The X-ray diffraction (XRD) measurements were performed with X'Pert-Pro MPD with Cu Kα (λ = 1.5406 Å). The surface morphologies of the polymer were determined by transmission electron microscopy (TEM) observed on Tecnai G2 F20 S-Twin (FEI, 200 kV). Scanning electron microscopy (SEM) images and energy-dispersive X-ray spectroscopy (EDX) was taken on Quanta 400FEG (FEI). Chemical compositions and element binding energies were analyzed using X-ray photoelectron spectroscopy (XPS) on Perkin-Elmer RBD upgraded PHI-5000C ESCA system with Al Kα x-ray as an excitation source. Raman spectra were collected using a Jobin-YvonLabRam HR 800 confocal micro-Raman system equipped with an electrically-cooled detector. The excitation wavelengths were 532 nm with a Nd:YAG laser. Thermogravimetric analysis (TGA) was conducted on EXSTAR TG/DTA 6200 (Seiko Instruments Inc.) at 25–1000°C in a 30 ml min^−1^ N_2_ flow with a rate of 5°C min^−1^. In this work, different electrodes were prepared for electrocatalytic experiments under the same situations.

### Electrode preparation

To prepare the working electrodes, 2 mg of the N-doped graphene samples were dispersed in a solution containing 1.8 ml of deionized water (18.2 MΩ) and 0.2 ml of 5 wt% Nafion aqueous solution. The mixture was ultrasonicated for 15 min to obtain a homogeneous catalyst ink. 15.00 μL of 1 mg mL^−1^ N-doped graphene dispersions was dropped onto a mirror polished glassy carbon electrode and dried in air at 60°C. Pt/C(20 wt%) was deposited on the electrode under the same procedure.

### Electrochemical test

Electrochemical measurements were performed using an Autolab PDSTAT302N electrochemical station with a typical three-electrode cell equipped with gas flow systems. Ag/AgCl and Pt wire were selected as reference electrode and the counter electrode, respectively. An aqueous solution of KOH (0.1 M) was used as electrolyte for both normal cyclic voltammogram and rotating ring-disk electrode (RRDE) voltammogram measurements. Before every test, an N_2_/O_2_ flow was used through the electrolyte for at least 30 min. Normal cyclic voltammograms were performed from 0.2 V to −1.0 V with a sweep rate of 50 mV s^−1^. For the rotating ring-disk electrode (RRDE) test, the same amount of catalyst was loaded on a rotating ring-disk electrode. The polarization curves for ORR were conducted with a scan rate of 10 mV s^−1^ at different rotating speeds from 600 to 2500 rpm from 0.2 to −1.2 V (vs Ag/AgCl).

The electron transfer number (n) for can be calculated by Koutecky-Levich equations as follows: 
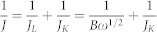

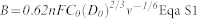

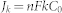


Where J_K_ is the kinetic current density, J_L_ is the diffusion–limiting current density, J is the measured current density. B is the reciprocal of the slope, ω is angular velocity of the disk (ω = 2*πN*). N is the linear rotation speed. F is the Faraday constant, *C*_0_ is the saturated concentration of O_2_ in 0.1 M KOH at room temperature, *D*_0_ is the diffusion coefficient of oxygen in water, *v* is the kinematic viscosity of the solution at room temperature.

### Theoretical method

In order to further understand the N doping effect in graphene on enhancing ORR performance, the density functional theory (DFT) calculations have been done in the VASP code^42^. The frozen-core all-electron projector-augmented wave (PAW) method was used^43^ with the Perdew-Burke-Ernzerhof approximation to the exchange-correlation functional^44^. The energy cutoff for the plane-wave expansion was set to 400 eV. Conjugated gradient (CG) atomic optimization is performed with a criterion of convergence of 0.01 eV/Å. A 2 × 2 ***k***-point grid was chosen in the Brillouin zone integration for a 8 × 8 super cell of graphene including 128 carbon atoms, and spin-polarized DFT were used in all calculations.

## Author Contributions

W.C. planed and supervised the project, Y.W.Z. synthesized and characterized samples, as well as conducted the ORR experiments. D.H.W. discussed the results and commented on the manuscript. J. G. conducted the ORR experiments. L.W., F.D. and X.M.T. designed the CN structural model and conducted theoretical calculations.

## Supplementary Material

Supplementary Information Supplementary Information
