# Supplementary material for: Manageable N-doped Graphene for High Performance Oxygen Reduction Reaction
Source: Sci Rep. 2013 Sep 26;3:2771. doi: 10.1038/srep02771 (PMC3783894; doi:10.1038/srep02771)
Supplement: Supplementary Information [file srep02771-s1.doc]

Supplementary Information

Manageable N-doped Graphene for High Performance Oxygen Reduction Reaction
Yuewei Zhang1, Jun Ge1, Lu Wang2, Donghong Wang1, Feng Ding3, Xiaoming Tao3 and Wei Chen*1
1 i-Lab, Suzhou Institute of Nano-Tech and Nano-Bionics, Chinese Academy of Sciences, Suzhou 215123, P. R. China, 2 Department of Applied Physics, The Hong Kong Polytechnic University, Kowloon, Hong Kong SAR, P. R. China, 3 Institute of Textile and Clothing, The Hong Kong Polytechnic University, Kowloon, Hong Kong SAR, P. R. China. 


T1= 550¡æ, T2=700¡æ, 800¡æ, 900¡æ

Figure S1. Process of preparation N-doped graphene. Glucose and another N-rich precursor (urea or melamine) go through a two-step thermal treatment according to the process.

sample	Mass ratio of two precursors	T1	T2	sample	Mass ratio of two precursors	T1	T2	
NCU5	m(Urea)/
m(Glucose)
=20	550	None	NCM5	m(Melamine)/
m(Glucose)
=20	550	None	
NCU57			700	NCM57			700	
NCU58			800	NCM58			800	
NCU59			900	NCM59			900	

Table S1. All the samples are denoted as NCXyz listed here.


Figure S2. Typical TEM images of NCU5 series.(a) NCU5, (b) NCU57, (c) NCU58, (d) NCU59. Scale bar for a-d is 50nm, 200 nm, 100nm, 50nm. 


Figure S3. Typical SEM images of N-doped graphene. (a) NCM5 and (b) NCU5 present coral-like structure, (c) NCM58 and (d) NCU58 are much looser and show silk-like structure. Scale bar is 200ìm, 5ìm, 5ìm and 2ìm respectively.


Figure S4. Structure characterizations of N-graphene derived from glucose and urea. (a)XRD patterns.(b)Thermo Gravimetric Analyzer (TGA) measurements. (c) Raman spectra of different samples.

XRD patterns reveal that by substituting urea for melamine, distinct intermediateproducts emerge from heating process. In Fig. 2a, we can tell that the involvement of glucose has little effect on the polymerization of melamine in the pre-treatment step. Most of NCM5 are made up with g-C3N4. However, when using urea as precursor, the formation of g-C3N4 is hindered. Fig. S4a shows that XRD pattern of NCU5hastwo obvious and broad peak at 2è=13.5° and 26.7°. Although the peak positions possess typical g-C3N4 characteristic, the ratio of I13.5°/26.7° is larger in comparison with g-C3N4, meaning the decreasing ordering of lamellar structure of carbon nitride, indicating that the polymerization process of urea under 550¡æproceeded incompletely. After deeper high-temperature treatment, the peak at 2è=26.7° of NCU5 series weakens, and the intensity of peak at 13°-14° gradually enhances. 
From the analysis of XRD results, it is obvious that different combinations of precursors lead to similar XRD patterns of final products, however, they go through featured process. The addition of glucose has an apparent impact on the polycondensation reaction of urea. The reason lies in the complicated path from urea to g-C3N4 and its low productivity. Glucose acts as an impediment parameter and results in even lower productivity and lower degree of polymerization. This blocking effect is not observed in melamine series since melamine is a high efficient precursor for g-C3N4 comparatively, thus glucose has little effect on the polymerization process.
Thermo Gravimetric Analyzer (TGA) results show that urea derived carbon nitride(UCN) decomposes completely before 700¡æ. As for NCU5, the residual mass percent reaches a platform after 920¡æat 4.5%. The huge weight lossmeans that NCU5constitutesunstable carbon nitridemostly, as well as some high carbon products. After treating with second-step high temperature, thermal stability of the products is much enhanced, indicating stable C-C bonds accounts for more proportion with the increase of temperature. About 50%mass of NCU59left evenconducting at 1000¡æin N2flow. 

Figure S5. XPS spectra of C1s, N1s and O1s for NCM5.

The N 1s spectrum can be fitted into four binding energies. Two main signals at 398.4 eV and 399.4 eV are exhibited. The former peak shows occurrence of C-N=C groups (denoted as N1). The second peak relate to either tertiary nitrogen N-(C)3 groups linking structural motif (C6N7) or amino functions carrying hydrogen ((C)2-N-H) in connection with structural defects and incomplete condensation (denoted as N2). A weak peak at 400.8 eV corresponds to N bonded three carbon atoms in the aromatic cycles. The 404.2 eV peak is attributed to the ð-excitations. The spectrum match the characteristic spectrum of g-C3N4. Thus it is reasonable to consider that most of the materials is made up with g-C3N4.

Figure S6. Elemental composition results from EDX for NCU5 and NCM5 series.


Figure S 7. XPS spectra of C1s, N1s and O1s for NCU5.

The N spectrum in Fig S6(b) is quite different from that of NCM5. Five peaks can be seperated here which are identified by the bonding state of the N atom in the composite. These peaks at 397.9, 399.5, and 403.5 eV refer to several N-containing functional groups. The peak at 397.9 eV relates to C-N=C groups exsited in graphitic carbon nitride and a small quantity of pyridinic nitrogen in N-doped carbons. The peak at 399.5eV is either tertiary nitrogen N-(C)3 groups linking structural motif (C6N7) of graphitic carbon nitride or amino functions carrying hydrogen ((C)2-N-H) located at the edge. The 404.3 eV peak is attributed to the ð-excitations as well. As discussed, N spectrum here presents distinctive characters compared to Fig S5(b). It can be regarded as combination of urea-derived and glucose-derived products. It is worth noting that urea-derived materials here no longer play a predominant role because of its low productivity.


Figure S8. Elemental analysis from XPS of NCU57, NCU58 and NCU59.


Figure S9. Elemental analysis of NCU57, NCU58 and NCU59. Highresolution C1s XPS spectra of (a) NCU57, (b)NCU58 and (c)NCU59. Highresolution N1s XPS spectra of (d) NCU57, (e) NCU58 and (f)NCU59


Figure S10. EDX mapping scan of NCU58 and NCM58.Scanning electron microscopic energy-dispersive X-Ray mapping images of NCU58 and NCM58demonstrate uniform distribution of C(red), N(green) and O(blue) atoms.


Figure S11. Cyclic voltammetry (CV) measurements. Featureless curves of CV in 0.1M KOH solution with N2 saturated for NCM57, NCM58, NCM59and graphene.


Figure S12. Linear sweep voltammetry (LSV) measurements.(a) NCM57, (b)NCM58, (c)NCM59,(d)Pt/C(20%). Koutecky-Levich plots at different potential for (e) NCM57 (f) NCM59.


Figure S13. Content curves of total N and ORR activities at -0.9V for NCU57, NCU58, NCU59.


Figure S14. Analysis of different N speciesfrom N1s spectra of NCM57, NCM58 and NCM59.


Figure S15. Cyclic voltammetry (CV) measurements. CV curves in 0.1M KOH solution with (a)N2 and (b)O2saturated for NCU57, NCU58 and NCU59.


Figure S16. Linear sweep voltammetry (LSV) measurements.RRDE voltammograms at different rotating rates and Koutecky-Levich plots at different potential for(a,d) NCU57, (b,e)NCU58,(c,f) NCU59.
Samples
(N%)	Structural Models	Spin density distribution	Maximum charge transfer (Charge transfer>0.15)	
NCM59
(8%)			0.18 (16%)	
NCU59
(12%)			0.18 (21%)	
NCU58
(25%)			0.31 (40%)	
NCM58
(25%)			0.34 (41%)	
NCU57
(33%)			0.44 (65%)	
Figure S17.Structural models and calculation results. Structural models with different N content and spin density distribution on the electron density isovalue plane (the positive value is yellow while the negative value is blue), together with the maximum charge transferof C atoms and the percentage of C atoms with charge transfer>0.15 in the parenthesis.
